# Sodium-Glucose Cotransporter 2 Inhibitors Mechanisms of Action: A Review

**DOI:** 10.3389/fmed.2021.777861

**Published:** 2021-12-20

**Authors:** Jorge I. Fonseca-Correa, Ricardo Correa-Rotter

**Affiliations:** Department of Nephrology and Mineral Metabolism, Instituto Nacional de Ciencias Médicas y Nutrición Salvador Zubirán, Mexico City, Mexico

**Keywords:** SGLT2 inhibitors, gliflozins, kidney disease, heart failure, diabetes

## Abstract

Sodium-Glucose Cotransporter 2 inhibitors (SGLT2i), or gliflozins, are a group of antidiabetic drugs that have shown improvement in renal and cardiovascular outcomes in patients with kidney disease, with and without diabetes. In this review, we will describe the different proposed mechanisms of action of SGLT2i. Gliflozins inhibit renal glucose reabsorption by blocking the SGLT2 cotransporters in the proximal tubules and causing glucosuria. This reduces glycemia and lowers HbA_1c_ by ~1.0%. The accompanying sodium excretion reverts the tubuloglomerular feedback and reduces intraglomerular pressure, which is central to the nephroprotective effects of SGLT2i. The caloric loss reduces weight, increases insulin sensitivity, lipid metabolism, and likely reduces lipotoxicity. Metabolism shifts toward gluconeogenesis and ketogenesis, thought to be protective for the heart and kidneys. Additionally, there is evidence of a reduction in tubular cell glucotoxicity through reduced mitochondrial dysfunction and inflammation. SGLT2i likely reduce kidney hypoxia by reducing tubular energy and oxygen demand. SGLT2i improve blood pressure through a negative sodium and water balance and possibly by inhibiting the sympathetic nervous system. These changes contribute to the improvement of cardiovascular function and are thought to be central in the cardiovascular benefits of SGLT2i. Gliflozins also reduce hepcidin levels, improving erythropoiesis and anemia. Finally, other possible mechanisms include a reduction in inflammatory markers, fibrosis, podocyte injury, and other related mechanisms. SGLT2i have shown significant and highly consistent benefits in renal and cardiovascular protection. The complexity and interconnectedness of the primary and secondary mechanisms of action make them a most interesting and exciting pharmacologic group.

## Introduction

Sodium Glucose Cotransporter 2 inhibitors (SGLT2i), also known as gliflozins, are an exciting and highly interesting group of “relatively new drugs” that have shown consistent positive results in renal and cardiovascular protection. They inhibit the action of the Sodium Glucose Cotransporter 2 (SGLT2) in the kidney and cause glucosuria. Initially, they were thought of and developed as glucose lowering therapies, yet large clinical trials in a very short time have demonstrated clinical benefits that far exceeded what was expected. In this short review we describe the physiologic effects of SGLT2 inhibitors and discuss the clinical benefits demonstrated to date.

## Physiology of SGLT2 Cotransporters and Renal Glucose Handling

SGLT2 cotransporters are part of a large family of symporters responsible for facilitated transport of different solutes, aided by a positive sodium gradient (1, 2). There are two main sodium-glucose cotransporters in the body: SGLT1 and SGLT2. SGLT2 cotransporters are almost exclusively found in renal tissue, whereas SGLT1 are mostly found in the small intestine, heart, and skeletal muscle, aside from the kidney (Table 1) (3–5). In the kidneys, SGLT2 and SGLT1 handle sodium and glucose reabsorption in the proximal tubules of the nephron. Their physiological function is to reabsorb 100% of filtered glucose, avoiding energy loss through glucosuria. SGLT2 cotransporters are found in the brush border of the renal tubular cells in the first segments of the proximal tubules (S1 and S2). They have a high transport capacity but low affinity for glucose and are responsible for the reabsorption of 90 to 97% of filtered glucose. The remaining 3–10% of filtered glucose is absorbed by the high-affinity, low-capacity, SGLT1 cotransporter, present in the S3 segment of the proximal tubule. Glucose exits these tubular cells back into the circulation through the GLUT2 (for cells with SGLT2) and GLUT1 (for cells with SGLT1) transporters in the basoletaral membrane. This unidirectional transport of glucose and sodium is coupled to, and maintained by, the Na-K-ATPase pump in the basolateral membrane.

**Table 1:** Main differences between SGLT2 and SGLT1 cotransporters.

	**SGLT2**	**SGLT1**
Gene	*SCL5A2* (16p11.2)	*SLC5A1* (22p13.1)
Tissue	Kidney^*^ (To a lower extent: brain, liver, thyroid, pancreas, skeletal muscle).	Small intestine^*^, heart, skeletal muscle, trachea, kidney, brain, testicles and prostate.
Selectivity	Glucose	Glucose, galactose
Capacity/affinity for glucose transport	High/Low	Low/High
Na:Glucose transport stoichiometry	1:1	2:1
Physiological function	Reabsorption of renal filtered glucose	Intestinal absorption of glucose and galactose; reabsorption of renal filtered glucose
Percentage of renal glucose reabsorption	90–97%	3–10% (up to 40% with SGLT2 inhibition)

* *Main location*.

*Adapted from references (3–5)*.

The coupled work between the high-capacity/low-affinity SGLT2 and low-capacity/high-affinity SGLT1 cotransporters handles the full load of filtered glucose. This way, in a normal physiological setting, glucose reabsorption by the proximal tubules is adjusted to the variations in serum glucose concentrations. Total glucose reabsorption is directly proportional to the amount of filtered glucose. This reabsorption capacity has a natural limit (Tmax_G_) that is reached when filtered glucose approximates 350 mg/min/1.73 m^2^, equivalent to between 180 and 200 mg/dL of glycemia. Glucosuria develops when hyperglycemia exceeds this Tmax_G_. In chronic hyperglycemia of diabetes, the kidney shifts the Tmax_G_ to higher glucose levels, around 240 mg/dL (6). The proximal tubules increase the number of SGLT2 cotransporters to make up for the increase in luminal glucose flow (7). This increase in SGLT2 cotransporters comes at a cost of energy expenditure through the basolateral Na-K-ATPase, and is thought to be central to the pathophysiology of diabetic kidney disease (8).

## SGLT2 Cotransporter Inhibitors Mechanisms of Action

The first SGLT2 inhibitor was named phlorizin, a naturally occurring phenolic glycoside derived from the root bark of the apple tree (9). It was first isolated in the 19th century and was originally thought to have antipyretic properties. Further analysis found that phlorizin causes glucosuria and it was thought to cause a diabetes-like state when administered to dogs, due to the presence of glucosuria, polyuria and weight loss.

With the characterization of renal glucose reabsorption in the proximal tubule in the 1960's, the cloning of the SGLT2 cotransporter in the 1990's (10), and further understanding of renal handling of glucose and the pharmacological effects of phlorizin, inhibition of renal glucose reabsorption was studied as a target for diabetes control. Preclinical studies in the 1980's showed that phlorizin improved insulin sensitivity in diabetic rat models without affecting insulin action in control rats (11).

Phlorizin has no oral bioavailability, so it can only be administered intravenously. The first orally available SGLT2 inhibitor, T-1095, was developed in the 1990's. It showed some improvement in HbA1c, reduction of microalbuminuria, and weight loss in rats (12, 13). Unfortunately, T-1095 was not selective to SGLT2 and its action on intestinal SGLT1 caused significant gastrointestinal adverse effects and intolerance. Following T-1095, at least seven different orally available SGLT2 inhibitors have been developed (2), three of which have been approved for use by the FDA: dapagliflozin, empagliflozin, and canagliflozin. The three of them are highly selective for SGLT2 inhibition over SGLT1.

Several clinical trials with empagliflozin (14–17), canagliflozin (18, 19), and dapagliflozin (20–22) in the past few years have demonstrated impressive benefits from SGLT2 inhibition in high-cardiovascular-risk patients. They have shown significant reduction of cardiovascular and all-cause mortality, hospitalizations for heart failure, adverse cardiovascular events, and progression of albuminuria, when added to standard therapy in diabetic and non-diabetic kidney disease. Particularly interesting is the fact that the use of SGLT2 has proven beneficial for kidney disease and heart failure despite the absence of diabetes as a central pathology. Understanding the direct and indirect physiological mechanisms and effects of SGLT2 inhibition is crucial to clarify why they offer a diversity of clinical benefits.

### Direct Physiological Effects of SGLT2 Inhibition

#### Glucosuria: Improvement in Glucose Control

Inhibition of SGLT2 cotransporter causes glucosuria. By inhibiting the SGLT2 cotransporter, gliflozins avoid glucose reabsorption in the S1 and S2 segments of the proximal tubule. This causes a reduction in Tmax_G_ to around 40–80 mg/dL (6) and a reduction in the renal threshold for glucosuria. To avoid significant energy loss through glucosuria, SLGT1 cotransporters compensate by increasing reabsorption to ~40% (23). A preclinical study in rats demonstrated this by showing that double SGTL1/SGLT2-knockout mice have significantly higher glucosuria than single SGLT2-knockout mice (24). Furthermore, glucose control from SGLT2 inhibition is not significantly associated with a higher risk of hypoglycemia (25).

Glucose control improvement is reflected by a reduction in HbA_1c_ of ~0.5–1% (14, 18, 19, 25, 26). This leads to an increased sensitivity to insulin and enhanced beta-cell function (27–30), and is of relevance in the role of SGLT2 inhibition in diabetes control. All the main clinical trials on SGLT2 inhibitors (14–16, 18–22) have demonstrated a consistent benefit in glucose control. The benefit is evident even when added to standard therapy (31).

#### Natriuresis: Improvement in Blood Pressure and Reversal of Tubuloglomerular Feedback Stimulation

Together with glucosuria, SGLT2 inhibition causes natriuresis that is associated with a negative salt and water balance (32). This reduction in plasma volume is evidenced by a drop in blood pressure of 3–6 mmHg in systolic and 1–1.5 mmHg in diastolic (18, 19) blood pressures.

Increased natriuresis and sodium delivery to the distal nephron is central for renal protection, as it normalizes the tubuloglomerular feedback mechanism. Chronic hyperglycemia of diabetes induces a state of increased reabsorption in the proximal tubule by increasing the SGLT2 cotransporter expression [and the Tmax_G_ (6)]. This increased glucose and sodium reabsorption reduces the delivery of sodium to the juxtaglomerular apparatus, stimulating the tubuloglomerular feedback, which in turn causes dilation of the afferent arteriole trying to “normalize” distal sodium delivery. Dilation of the afferent arteriole increases intraglomerular pressure and causes hyperfiltration, characteristic of diabetic kidney disease. SGLT2 inhibition reverses this feedback loop by increasing sodium delivery to the juxtaglomerular apparatus, inhibiting the tubuloglomerular feedback and causing constriction of the afferent arteriole (6, 33). The result is a reduction in intraglomerular pressure and improvement of hyperfiltration, that is reflected as an initial drop of glomerular filtration rate (GFR). This drop in GFR is reversible when SGLT2 inhibition is discontinued and is a response to hemodynamic changes. Although this initial GFR drop associated to SGLT2 inhibition may seem significant, its magnitude is limited in most clinical instances to 2–4 ml/min. Studies with long term follow up show that it is not continuous and is significantly less than the eGFR decline observed in the placebo groups.

### Secondary Effects of SGLT2 Inhibition

#### Improvement in Albuminuria

Clinical trials on diabetic and non-diabetic patients with chronic kidney disease (CKD) (22, 34) have demonstrated that SGLT2 inhibitors reduce albuminuria significantly. This effect is independent and additive to the effect of RAAS blockade (15). The improvement in albuminuria is multifactorial, related to the vasoconstriction of the afferent arteriole, the subsequent reduction in intraglomerular pressure and hyperfiltration, as well as the improvement in systemic blood pressure.

Some studies have also suggested that podocytes benefit from SGLT2 inhibition, as they have SGLT2 cotransporters, and the use of dapagliflozin or empagliflozin reduces podocyte dysfunction and effacement (35, 36) through normalization in insulin sensitivity and improvement in glucotoxicity. This would lead to an improvement in albuminuria (37).

#### Weight Loss and Lipid Metabolism Shift

The use of SGLT2 inhibitors induces weight loss of between 2 and 4 kg after 6–12 months of treatment (25, 26, 29, 38–41). Initial weight loss is related to volume contraction and subsequently secondary to caloric wasting through glucosuria. ADA guidelines actually recommend SGLT2 inhibitors as initial antidiabetic therapy when weight loss is desired as part of the treatment (42).

SGLT2 inhibition and the subsequent glucosuria induces a state of relative glucose “deprivation,” shifting energetic substrate use to lipids. This reduces cellular lipotoxicity and improves oxidative stress. This also favors an increase in ketone production, which appear to be a better energetic substrate for renal and myocardial cells.

#### Improvement in Proximal Tubular Work and Oxygen Consumption

As described previously, hyperglycemia of diabetes induces a state of glucose and sodium hyperreabsorption in the proximal tubule, activates tubuloglomerular feedback and causes hyperfiltration. This positive feedback loop increases cellular work due to increased Na-K-ATPase activity and induces proximal tubular hypertrophy. Aside from this, increased intracellular glucose in proximal tubular cells is diverted to non-glycolytic pathways increasing advanced glycation end-products, affecting mitochondrial activity, and increasing oxidative stress. Inhibition of the tubuloglomerular feedback, hyperfiltration and increased glucose reabsorption reduces energy expenditure and oxygen consumption in proximal cells (8). Reducing serum and intracellular glucose levels, reduces cellular glucotoxicity.

#### Improved Oxygen Delivery and Anemia

The improvement in proximal tubular cell work and reduction in energy expenditure described above, reduces oxygen demand and increases cortical oxygen tension (8, 43). Subsequent delivery of glucose to the latter part of the proximal tubule is reabsorbed by the SGLT1 cotransporters and increases energy expenditure and oxygen consumption in the renal outer medulla (44–46). The decrease in oxygen availability stimulates hypoxia-inducible factors HIF1 and HIF2 (47) and enhance the release of erythropoietin (48). This, together with a mild volume contraction, increases the hemoglobin and favors oxygen delivery to different tissues. Clinical trials have shown improvement in hemoglobin levels in patients treated with SGLT2i (49). Dapagliflozin appears to suppress hepcidin and other iron-metabolism related proteins, helping improve erythropoiesis (50).

### Other Possible Effects

Gliflozins appear to reduce inflammatory markers such as IL-6, TNF, IFNγ, NF-κβ, TLR-4, and TGF-β (51–54). They also appear to improve mitochondrial function (55) reduce mesangial expansion and the number of myofibroblast in myocardial tissue (56). Empagliflozin appears to reduce IL-β inflammatory pathway in proximal tubular cells (57). These effects would reduce inflammation, fibrosis, and oxidative stress in myocardial and renal tissue. Nevertheless, all these changes appear to be secondary to the metabolic and hemodynamic effects of SGLT2 inhibition.

### Adverse Effects Associated to SGLT2 Inhibition

The use of SGLT2 inhibitors is associated with adverse events that are rare and generally mild, yet should be considered. Volume contraction and osmotic diuresis are direct effects of their mechanism of action and may infrequently present of a magnitude of significance when the drug is initiated in geriatric patients and in those on diuretics. In some clinical trials, a particularly relevant yet infrequent, adverse effect described was euglycemic diabetic ketoacidosis, yet this was not present in CREDENCE and DAPA-CKD. Finally, genital mycotic infections are up to four times more frequent in patients using SGLT2 inhibitors. They are generally mild and easily treatable. Patients should be counseled to monitor signs and symptoms and to maintain adequate genital hygiene. Other adverse events such as bone fractures and amputations appeared to be associated to SGLT2 inhibition in the CANVAS trial, but they have not been replicated in other studies.

## Clinical Benefits of SGLT2 Inhibition

Several clinical trials have demonstrated important cardiovascular and renal benefits of SGLT2 inhibition. The EMPA-REG OUTCOME (14) trial was the first study to demonstrate this in a large scale. Published in 2015, this double-blind, multicenter, clinical trial was aimed at demonstrating cardiovascular safety of empagliflozin when added to standard therapy in high cardiovascular risk diabetic patients with an estimated glomerular filtration rate (eGFR) ≥30 ml/min/1.73 m^2^. After a 3 year follow up, empagliflozin not only proved to be safe, but improved major cardiovascular events (cardiovascular death, myocardial infarction, or stroke) by 14%, reduced all-cause mortality by 32%, cardiovascular death by 37%, and hospitalization due to heart failure by 35%. A subsequent analysis of secondary outcomes (15) confirmed that empagliflozin reduced incident or worsening diabetic nephropathy by 39%. Baseline eGFR declined in subjects on both the placebo and empagliflozin arms, yet this decline in eGFR stabilized after a few weeks in the empagliflozin group and was reverted after empagliflozin was discontinued, showing that the change in eGFR is not related to kidney injury but to hemodynamic changes induced by the drug itself. These results were notable considering that renal outcomes were not the primary outcome of the trial and 80% of subjects were already under RAAS blockade as standard therapy for diabetic kidney disease.

Similarly, the CANVAS (18) trial, in 2017, demonstrated that canagliflozin added to standard therapy in high cardiovascular risk patients reduced mayor cardiovascular events by 14%, cardiovascular death by 13%, myocardial infarction and stroke by 14 and 10% respectively, and hospitalization for heart failure by 33%. Subjects on canagliflozin had a 40% lower risk of adverse renal outcomes (renal function decline, dialysis initiation or death from a renal cause), 27% lower risk of worsening albuminuria and a 1.7 higher likelihood of improvement in albuminuria.

In 2019, the DECLARE-TIMI 58 (20) trial, which included over 17,000 patients followed for 4.2 years, showed that dapagliflozin reduced the risk of hospitalization for heart failure by 27% and of adverse renal outcomes by 27%. The large population of diabetic patients included in this trial represents the widest range of renal function in any of the cardiovascular outcome studies.

An interesting meta-analysis (58) of these trials show that SGLT2 inhibitors significantly reduced the risk of kidney failure by 29%, end-stage kidney disease by 32% and acute kidney injury by 25%. These benefits were consistent across the different subgroups of GFR and albuminuria. Altogether, these studies demonstrate cardiovascular and renal benefit from SGLT2 inhibition in patients with diabetes and high cardiovascular risk, with and without established diabetic nephropathy. Another meta-analysis (59) of the EMPA-REG OUTCOME, CANVAS and DECLARE-TIMI 58 trials stratified the subjects (*n* = 34,322) according to eGFR and demonstrated that SGLT2 inhibitors had a better effect in reducing adverse renal outcomes (worsening renal function, ESKD or death from renal cause) when eGFR is between 30 and 60 ml/min/1.73 m^2^.

The CREDENCE (19) trial, published in 2019, was the first designed to focus on a composite renal outcome that included end stage kidney disease (ESKD) (dialysis requirement, kidney transplantation or eGFR of <15 ml/min/1.73 m^2^), doubling of serum creatinine or renal or cardiovascular death. Similar to CANVAS and EMPA-REG OUTCOME, subjects were diabetic, yet patients in this study had eGFR between 30 and 90 ml/min; 60% of which had to have an eGFR between 30 and 60 ml/min, and all subjects had urinary albumin-creatinine ratio (UACR) between 300 and 5,000 mg/g and optimal renin angiotensin system (RAAS) inhibition. Canagliflozin was associated with a significantly lower risk of adverse renal and cardiovascular outcomes, and the results were so evident and encouraging that, after interim analysis, the trial was stopped prematurely after 2.6 years of follow-up, displaying a reduction of 34% of the primary composite outcome in the canagliflozin group. A secondary analysis (60) of patients in the CREDENCE trial demonstrated that subjects receiving canagliflozin had a lower risk of renal and cardiovascular outcomes even when starting treatment with eGFR between 30 and <45 ml/min/1.73 m^2^.

In 2020, the DAPA-CKD (22) study was published. It included over 4,000 CKD patients, comprised 68% by diabetics and 32% by patients with CKD not related to diabetes, with an eGFR of 25–75 ml/min/1.73 m^2^ and UACR of 200–5,000 mg/g, treated with dapagliflozin or placebo. The study, as CREDENCE, was stopped prematurely due to the clear benefit offered by dapagliflozin in both diabetic and non-diabetic patients with CKD. The primary renal outcome (sustained reduction of at least 50% of eGFR, ESKD or renal or cardiovascular death) was significantly lower (HR: 0.61; 95% CI: 0.51–0.72; *p* < 0.001) in dapagliflozin treated patients. Benefits were also independent of the baseline presence of cardiovascular disease (61). A prespecified subanalysis (62) of subjects with eGFR <30 ml/min/1.73 m^2^ showed that dapagliflozin is safe and effective even in this lower eGFR levels. Similarly, the subgroup of patients with IgA nephropathy (63) treated with dapagliflozin had a lower risk of kidney disease progression, with similar safety profiles when compared to placebo.

There have been important studies to explore of the effects of SGLT2 inhibition on patients with high cardiovascular risk, with or without kidney disease and independently of the presence of diabetes. The DAPA-HF (21) and the EMPEROR-reduced (16) trials demonstrated that the use of either dapagliflozin or empagliflozin reduces cardiovascular death and worsening of heart failure in patients with heart failure and reduced ejection fraction, regardless of the presence or not of diabetes. More recently the EMPEROR-preserved (17) trial also demonstrated similar benefits from empagliflozin in patients with heart failure and preserved ejection fraction.

## Conclusions

The positive results discussed above have been clearly striking and consistent, demonstrating a significant improvement in cardiovascular and renal outcomes by the different SGLT2 inhibitors, when added to optimized standard therapy that includes maximal RAAS inhibition.

The cascade of events induced by the inhibition of SGLT2 cotransporters has proven beneficial to reduce cardiovascular and renal outcomes, and death in patients with and without diabetes. The exact mechanisms of cardiovascular as well as renal benefits are probably related to multiple interplaying factors, but are not completely understood (64). These include a reduction in glycemia with subsequent improvement in insulin resistance, weight loss and reduced visceral fat. Correction of glycemia reduces direct glucotoxicity and has shown improvement in cellular function in proximal renal tubular cells as well as other tissues.

Although SGLT2 inhibition favors an improvement in HbA_1c_, the extent of this improvement is not enough to explain the significant clinical benefits observed in cardio-renal health. Similarly, hyperglycemia would not be a central pathophysiological issue in patients without diabetes. A possible energetic benefit is the shift to lipid metabolism, with a subsequent reduction in lipotoxicity, as well as an increase in ketone production. Central to the cardiovascular benefit is the diuretic and natriuretic effect of SGLT2 inhibition. The described improvement in volume status, sodium balance and blood pressure seem to be of relevance to both the cardiovascular as well as renal benefits. Reduction in albuminuria, inflammation and oxidative stress have also been implicated. In addition, for the renal component, the intrarenal hemodynamic mechanisms described seem to be key for the long-term improvement in the eGFR slope decline, in diabetic as well as non-diabetic patients.

## Author Contributions

JF-C and RC-R contributed equally to conception and initial discussions regarding the main subject matter, manuscript focus, and general outline. JF-C performed the initial literature review and drafted the first version of the manuscript and was in charge of writing the final manuscript. RC-R oversaw the structure and logical sequencing of the manuscript, perfected the drafting and ensured that the information was up to date, and added sections to the initial draft. Both authors read and approved the final submitted version.

## Conflict of Interest

The authors declare that the research was conducted in the absence of any commercial or financial relationships that could be construed as a potential conflict of interest.

## Publisher's Note

All claims expressed in this article are solely those of the authors and do not necessarily represent those of their affiliated organizations, or those of the publisher, the editors and the reviewers. Any product that may be evaluated in this article, or claim that may be made by its manufacturer, is not guaranteed or endorsed by the publisher.
